# Use of a participatory quality assessment and improvement tool for maternal and neonatal hospital care. Part 2: Review of the results of quality cycles and of factors influencing change

**DOI:** 10.7189/jogh.10.020433

**Published:** 2020-12

**Authors:** Giorgio Tamburlini, Alberta Bacci, Marina Daniele, Stelian Hodorogea, Dalia Jeckaite, Audrius Maciulevicius, Emanuelle Pessa Valente, Gelmius Siupsinskas, Fabio Uxa, Francesca Vezzini, Ornella Lincetto, Maurice Bucagu

**Affiliations:** 1 Centro per la Salute del Bambino, Trieste, Italy; 2 International perinatal care consultant, Trieste, Italy; 3 Department of Women and Children's Health, Faculty of Life Sciences and Medicine, King's College, London, UK; 4 Department of Obstetrics and Gynecology, State University of Medicine and Pharmacy, Chisinau, Moldova; 5 International midwifery and perinatal care consultant, Panevezys, Lithuania; 6 Neonatal Unit, Lithuanian University of Health Sciences, Vilnius, Lithuania; 7 Institute for Maternal and Child Health IRCCS Burlo Garofolo, WHO Collaborating Centre for Maternal and Child Health, Trieste, Italy and Instituto de Medicina Integral Fernando Figueira, Recife, Brazil; 8 International perinatal care consultant, Basel, Switzerland; 9 Institute for Maternal and Child Health IRCCS Burlo Garofolo, WHO Collaborating Centre for Maternal and Child Health, Trieste, Italy; 10 Instituto de Medicina Integral Fernando Figueira, Recife, Brazil; 11 WHO Department of Maternal, Newborn, Child, Adolescent Health and Ageing, Geneva, Switzerland; 12 WHO Department of Maternal, Newborn, Child, Adolescent Health and Ageing, Geneva, Switzerland

## Abstract

**Background:**

Information about the use of the findings of quality assessments in maternal and neonatal (MN) care is lacking and the development of tools capable to effectively address quality gaps is a key priority. Furthermore, little is known about factors that act as barriers or facilitators to change at facility level. Based on the extensive experience made with the WHO Quality Assessment and Improvement MN (QA/QI MN) tool, an overview is provided of the improvements in quality of care (QoC) which were obtained over time and of the factors influencing change.

**Methods:**

All documented reports on the implementation of the WHO QA/QI MN tool were searched and screened for inclusion. Reports were considered if bringing evidence from both the baseline assessment and the reassessment. Changes were considered in four domains: maternal care, neonatal care, infrastructure and policies, with reference made to WHO maternal and neonatal care standards. The observed improvements were categorized according to intensity and extent across the sample of health facilities. Factors influencing change were categorized into internal and external and further classified as barriers or facilitators.

**Results:**

Changes were documented after an average period of 1.2 years from first assessment in 27 facilities belonging to 9 different countries in Central and Eastern Europe (3), Central Asia (3), sub-Saharan Africa (2) and Latin America (1). Improvements were observed in all areas of care but were greater and more frequently observed in areas related to appropriate case management and respectful care for both mothers and newborns. Although widespread across most facilities and countries, the observed improvements were not covering all the quality gaps observed at the baseline assessment nor were always sufficient to achieve standard care. Factors facilitating change as well as barriers were mainly related to the capacity of the managers and head of units to involve and motivate their staff members.

**Conclusions:**

The use of WHO QA/QI MN tool proved effective in promoting significant changes in quality of care. The review of observed improvements and of factors influencing change at facility level shows that participatory assessment tools that promote a constructive dialogue with hospital managers and staff and support them in acquiring capacity in this role are crucial to implement effective quality cycles.

To respond to the widely documented gaps in maternal and neonatal hospital care (MNHC), international agencies and country health authorities have strengthened their efforts to improve quality through action at system as well as at facility level [1-5]. However, while quality assessments have been intensified using a variety of tools, there is still scarce evidence about the results of quality improvement cycles in MNHC over the whole continuum of care from admission to discharge [6-8]. A review of existing quality assessment tools focusing on MNCH highlighted that information about the use of the collected information for quality improvement, let alone about the results of action taken, was lacking and indicated the development of assessment tools capable to help addressing important quality gaps as a key priority for research [9]. Furthermore, while substantial progress was made in identifying factors that determine quality at system level little is known about factors that may represent barriers or facilitators to change at facility level [8,10]. This makes it difficult for national health authorities, international agencies, development partners and local managers to respond to the call to identify and implement effective approaches to improve the quality of MN hospital care and ensure adherence to WHO MN standards [11].

The experience with the WHO Quality Assessment and quality improvement maternal and neonatal (QA/QI MN) tool [12] is the largest documented so far in maternal and neonatal quality of care (QoC) assessment, as it covers 25 countries and a wide variety of health systems [13]. The results of the quality cycles initiated through this approach have been documented in published papers or in unpublished reports. A few of these assessments included also an analysis of factors influencing the observed changes in quality of care [14-16]. Based on this rich body of information, we made an overview of the improvements in QoC which were documented in subsequent assessments and of the factors influencing change.

## METHODS

### Inclusion and exclusion criteria

All documented reports on the implementation of the WHO QA/QI MN tool were first searched and then screened for inclusion in this review. They include papers published in peer reviewed journals, reports retrievable from WHO or other Agencies’ websites. In order to capture the changes in QoC, reports were considered if bringing evidence from both the baseline assessment and the reassessment after a period of time, with a detailed list of areas of care where changes were observed. Within this subset of reports, information related to factors influencing the observed changes was only considered if: a) resulting from an explicit attempt to capture this dynamic as a study objective; b) based on interviews, either face-to-face or through web-based forms with key staff and managers of the assessed facilities; and c) if factors could be identified as internal or external to the facility and acting as barriers or facilitators.

### Retrieval of information and methods for the analysis of QI improvements cycles

In order to ensure consistency with the way findings related to main quality gaps in MNHC as assessed by the WHO QA/QI MN tool were reported [13], the same framework was used to describe results. The framework differentiates four main components (maternal care, neonatal care, infrastructure and policies) with reference to WHO maternal and neonatal care standards [11]. The information retrieved from each report included country, reach of the assessment, year of the assessment and reassessment, involved authorities and agencies, composition of assessment team, list of observed changes in MNHC at reassessment.

The observed improvements were categorized according to intensity of change and the extent of the observed changes across the sample of health facilities. Within each report, observed improvements in quality of care were analysed for their intensity with reference to the scoring system used in the WHO QA/QI MN tool [12]. Intensity was considered moderate when the improvement observed in a specific area as compared to the baseline assessment was not sufficient to achieve a score corresponding to recommended standard care; substantial when it allowed to achieve a score corresponding to recommended standard care. To provide a semiquantitative idea of the extent of the observed changes, we used countries as unit of observation rather than facilities, as the number of facilities included for each country was quite variable. This approach led to four possible categories, which were used for reporting the evidence of the review:

1. moderate change in a minority of countries;
2. moderate change in a majority of countries;
3. substantial change in a minority of countries;
4. substantial change in a majority of countries.

This semiquantitative approach still allows to provide a measure of the observed changes and reduces the risk of over or under-representing the extension of improvement which would result from taking into account the facilities as unit of observation.

### Retrieval of information and methods for the analysis of factors influencing change

When the analysis of factors influencing change was available, data on the reported factors were extracted and factors were categorized into *internal* and *external*. The first category includes elements and dynamics internal to the facilities while the second includes factors depending on the outside environment (health system, and beyond). These factors were further classified as barriers or facilitators, ie, elements or dynamics that were hampering or driving quality improvement, and the information was ultimately classified into four categories: a) factors internal to the assessed facility that facilitated improvements; b) factors internal to the assessed facility that hampered improvements; c) factors external to the assessed facility that facilitated improvements; 4) factors external to the assessed facility that hampered improvements. Factors relevant to at least two countries were identified and reported. This analysis was made by two of the Authors (GT and AB).

## RESULTS

Overall, a reassessment was done and could be documented in 27 facilities belonging to 9 different countries, belonging to Central and Eastern Europe (3 countries), Central Asia (3), sub-Saharan Africa (2) and Latin America (1).

Time elapsing from first to the second assessment varied from around a year to almost 4 years. In all countries the reassessment was carried out by a national multidisciplinary team with the supervision of an international team [12,13]. In most countries the exercise was led by international agencies in collaboration with national authorities, in two by NGOs in collaboration with local authorities. In one the assessment was made within a project funded by a national research institution.

**Table 1** provides an overview of countries, dates, number of hospitals involved and organizing agencies.

**Table 1:** Countries where at least a first QA/QI cycle based on the WHO MN tool was completed

Country	Assessment and reassessment	Team	Number hospitals involved	Lead organization / partners
Albania	02/2009, 10/2011	International plus national	3	MoH/WHO/Spanish Government
Brazil	05/2015, 05/2016	International plus national	6	National Research Council, Pernambuco Health Authorities
Ethiopia	09/2012, 10/2016	International plus local	1	District Health Authorities/DwA CUAMM
Kazakhstan	11/2009, 04/2011	International plus national	4	MoH/WHO/European Union
Kyrgyzstan	03/2012, 05/2014	International plus national	3	MoH/WHO/UNFPA/UNICEF
Montenegro	12/2011, 01/2016	International plus national	3	MoH/UNICEF
Republic of Moldova, Transnistrian Region	11/2013, 07/2015	International plus national	2	MoH/WHO/Swiss Agency for Development and Cooperation
Tanzania	08/2012, 08/2016	International plus local	1	Regional and District Health Authorities/DwA Cuamm
Uzbekistan	04/2010, 04/2011	International plus national	4	MoH/UNICEF/WHO/EU

MoH – Ministry of Health, WHO – World Health Organization, DwA – Doctors with Africa

### Observed quality improvement

The following tables describe the improvements in the quality of MN care which were observed at reassessment as compared to baseline assessments. Results are framed according to maternal care, neonatal care, infrastructure and policies, each presented in a separate table (**Table 2****,** **Table 3****,** **Table 4** and **Table 5**). For each main area where improvements were observed, a semiquantitative description of the intensity and extent of the observed changes is given according to the four categories described in the Methods section. To provide a more detailed and granular description of changes, quotes directly taken from reports are reported.

**Table 2:** Intensity, extent and content of observed improvements in MN quality of care in nine countries related to provision of effective, safe and respectful care to mothers according to WHO Standards 1, 4, 5 and 6

WHO standard	Related areas	Observed improvements	
**Intensity and extent**	**Generic content** *(quotes from reports to provide examples)*
Standard 1: Every woman and newborn receives routine, evidence-based care and management of complications during labour, childbirth and the early postnatal period, according to WHO guidelines	Monitoring of maternal and foetal conditions during labour and birth	**+••**	Improved (more frequent, regular and recorded) monitoring of FHR and maternal conditions during labour and childbirth.
			*“Maternal and neonatal records were better filled in and vital signs recorded far more frequently”*
	Excess and/or inappropriate interventions	**++••**	Avoidance of many unnecessary /dangerous medications and interventions for healthy mothers and babies.
			Reduced use of unsubstantiated diagnostic categories.
			*“Routine procedures with unproven efficiency, such as enema, pubic shaving, catheterization, ice packs on abdomen after birth, were abandoned”*
			*“Percentage of episiotomies decreased substantially”*
			*“Non-existent diagnoses such as oedema of pregnancy were abandoned, and inappropriate routine restrictions, eg, fluid intake, were abolished”*
	Early identification and management emergencies	**+••**	Improved prevention (active management of 3rd stage of labour) and management of Post-Partum Haemorrhage.
			*“A team approach was noted in emergency situations”*
			*“The assessment of blood loss was far more accurate”*
	Management of clinical complications	**+••**	Management of selected obstetrical complications following international guidelines.
			*“Improved management of preterm labour (use of steroids and appropriate tocolytics when required)”*
			*“Misoprostol as last resort for treatment of atonic PPH and main method of labour induction”*
	Caesarean section indications and procedures	**+••**	Reduction of inappropriate indications for caesarean section.
			Increased use of epidural anaesthesia.
			*“Actions to address inappropriate interventions and the increasing caesarean section rate have been implemented, for example with adoption of Robson’s classification”*
			*“Increased accessibility to regional analgesia for CS”*
			*“Prophylactic administration of antibiotics was implemented to prevent infections after CS, instead of previous long (3-5 days) treatment courses”*
Standard 4: Communication with women and their families is effective and responds to their needs and preferences	Effective communication	**+••**	Improved written and oral information to pregnant women and mothers.
			*“Since previous assessment visual aids and printed information for women and families were developed and used (eg, at-risk pregnant women know when, where and to whom to seek the help in emergencies, have phones numbers of their health care providers)”*
			*“A discharge note for mothers with essential information on maternal and neonatal health issues was prepared”*
Standard 5: Women and newborns receive care with respect and preservation of their dignity	Respect and dignity	**++••**	Improved privacy at labour and birth.
			Choice of position in labour and birth by women is allowed and encouraged.
			*“Continuous support during labour and childbirth (food and drink intake, sympathetic attitude towards the patient) were observed”*
Standard 6: Every woman and her family are provided with emotional support that is sensitive to their needs and strengthens the woman’s capability	Emotional support	**++•**	Increased acceptance of companionship during labour and delivery.
			Encouraged and increased partner/father presence.
			*“A more friendly attitude towards women and their families by health providers was observed”*

CS – caesarean section, FHR – Foetal heart rate, PPH – post-partum haemorrhage, +• − moderate change observed in a minority of countries, +•• − moderate change observed in a majority of countries, ++• − substantial change observed in a minority of countries, ++•• − substantial change observed in a majority of countries

**Table 3:** Intensity, extent and content of observed improvements in MN quality of care in nine countries related to provision of effective, safe and respectful care to newborn babies according to WHO Standards 1 and 5

WHO standard	Related areas	Observed improvements	
**Intensity and extent**	**Generic content** *(quotes from reports to provide examples)*
Standard 1: Every woman and newborn receives routine, evidence-based care and management of complications during labour, childbirth and the early postnatal period, according to WHO guidelines	Early mother-baby contact and immediate initiation of breastfeeding	**+••**	Skin to skin contact after birth introduced or increased
			Increased initiation of breastfeeding within the first hour
			More widespread use of rooming-in
			Delayed cutting of the umbilical cord introduced
			Increased mothers’ participation in neonatal care, especially for sick newborns
			*“Improved practices in the delivery room were observed: prevention of hypothermia, immediate skin to skin contact, early breastfeeding, appropriate timing of umbilical cord cutting, transfer of mother and baby together to postpartum unit”*
			*“Breastfeeding support improved, including early enteral breast milk feeding for ill and pre-term newborns”*
			*“Examination of newborn by specialist is done in the presence of mother in mother and baby’s room”*
			*“Length of the separation baby and mother after CS is shortened from 6 to 24 hrs. in comparison with previous practice (48hrs)”*
	Resuscitation preparedness and procedures	**+•**	Improved readiness for newborn resuscitation.
			*“The supply of resuscitation equipment improved everywhere, the resuscitation pathways are available in two languages, as well as the mannequins for practical training, the knowledge on processing the equipment, theoretical knowledge of neonatologists, midwives, neonatal nurses were improved”*
	Care for premature / LBW babies	**++••**	Introduction of Kangaroo Care (KC) (training, guidelines and at least partial implementation)
			*“A room was identified to promote KC and nurses trained”*
	Excess and/or inappropriate interventions	**++•**	Decreased use of unnecessary drugs, diagnostics and reduced hospital stay
			*“Earlier discharge after birth of healthy women and newborns (within 3 days instead of previous 6)”*
			*“The use of drugs with unproven efficacy in the newborn was significantly reduced”*
	Early identification and monitoring of risk conditions and complications	**+••**	The identification and registration of hypothermia cases have improved
			Local protocols for complications developed based on international guidelines
			“*Advice given to mothers on baby’s dangerous signs”*
	Management of complications	**+••**	Improved indication and choice of antibiotics
			*“The follow up of sick, premature infants in NICU has substantially improved. Nurses are able to properly use equipment and monitor vital functions”*
			*“Drug provision has improved and laboratory diagnostics has improved”*
	Mother-baby bonding	**+••**	Improved skin to skin at birth and closer contact ensured after birth.
			*“Decreased separation even after CS, rooming-in is now the rule”*
			*“Involvement of mothers in care of sick newborns, including those in intensive care”*
Standard 5: Women and newborns receive care with respect and preservation of their dignity	Pain prevention and relief	**+•**	Reduced painful procedures and more attention paid to ensure a softer environment to newborns
			*“Blood sampling was reduced to minimum for sick newborn babies ”*
			*“Use of cell phone was prohibited in the NICU”*

CS – caesarean section, KC – kangaroo care, LBW – low birth weight, NICU – neonatal intensive care unit, +• − moderate change observed in a minority of countries, +•• − moderate change observed in a majority of countries, ++• − substantial change observed in a minority of countries, ++•• − substantial change observed in a majority of countries

**Table 4:** Intensity, extent and content of observed improvements in MN quality of care in nine countries related to human resources and infrastructure according to WHO Standards 7 and 8

WHO Standard	Related areas	Observed improvements	
**Intensity and extent**	**Generic content** *(quotes from reports to provide examples)*
Standard 7: For every woman and newborn, competent, motivated staff are consistently available to provide routine care and manage complications	Human resources number and skills mix	**+•**	Extension of clinical tasks for midwives and nurses
			Revised staff requirements for NICUs
			Increase in training opportunities.
			*“The role of nurses in the care of sick and premature newborns was enhanced, their skills improved, eg, their communication skills in dealing with mothers and their relatives”*
			*“After retraining in essential perinatal care, the midwives’ responsibilities were expanded”*
			*“The persisting shortage of neonatologists as well as anaesthesiologists was brought to the attention of national health authorities”*
			*“A request for establishing national standards regarding the number of midwives in maternity departments was made to improve the ratio of midwives to doctors”*
Standard 8: The health facility has an appropriate physical environment, with adequate water, sanitation and energy supplies, medicines, supplies and equipment for routine maternal and newborn care and management of complications	Hygienic facilities and waste disposal	**+••**	Improvement in the availability of hygienic facilities for pregnant women and mothers
			*“There was a general improvement in some basic amenities and services: availability of cold and warm water, toilets and basic supplies such as soap and antiseptics”*
			“*The waste disposal was improved*”
	Water, energy	**+••**	Improvement in the provision of basic services with implications for both effective care and patients’ comfort
			*“The areas which showed the greatest improvements were continuous water and energy availability”*
	Physical structure	**+••**	Improved pathways for emergencies
			Improved privacy ensured (eg, individual labour and delivery rooms or curtains used to separate beds)
			*“A dedicated area for the care of sick newborn babies was set up to allow for rooming-in, continuous and active presence of the mothers and availability of breast milk”*
	Essential equipment and supplies	**+•**	Improved maintenance of equipment and equipment maintained in good working order
			*“The emergency kits were organized and are present in every delivery room, where possible, or is mobile/easily accessible kit for two/three delivery rooms”*
			*“There have been improvements in the availability of basic supplies for laboratory”*
			*“Old equipment was replaced and renovated: delivery beds, overhead heaters, newborn resuscitation equipment”*
	Essential medicines	**+•**	Improved availability of essential drugs at different points of care (emergency, wards, delivery room, theatre).
			*“An emergency kit was now available even in the pre and postnatal wards”*

+• − moderate change observed in a minority of countries, +•• − moderate change observed in a majority of countries, ++• − substantial change observed in a minority of countries, ++•• − substantial change observed in a majority of countries, NICU − neonatal intensive care unit

**Table 5:** Intensity, extent and content of observed improvements in MN quality of care in nine countries related to policies according to WHO Standards 1, 2, 3 and 5

WHO standard	Related areas	Observed improvements	
**Intensity and extent**	**Generic content** *(quotes from reports to provide examples)*
Standard 1: Every woman and newborn receives routine, evidence-based care and management of complications during labour, childbirth and the early postnatal period, according to WHO guidelines	Infection prevention and control	**+••**	Improved infection prevention practices
			*“Since last assessment, a water tank for hand washing and hand sanitizer gel are available in every ward, as recommended by the WHO. Staff wash their hands often and use disposable gloves, changing them between one patient and another”*
			*“Inappropriate practices such as pre-operative shaving abolished”*
			*“In every delivery room (maternities included in assessment) scheme for correct hand washing is displayed on a wall close to the washstand”*
	National clinical guidelines and local protocols	**++•**	Development of local protocols based on national/international clinical guidelines
			“*Local neonatal protocols were developed: eg, hypothermia; pregnancy risk scoring was revised; indications for referral were established”*
Standard 2: The health information system enables use of data to ensure early, appropriate action to improve the care of every woman and newborn	Data collection and use	**++•**	Improved data collection and reporting
			*“Classification of neonatal death by birth weight and time of death was introduced”*
			*“A neonatal nursing record was developed”*
	Periodical perinatal audit	**+••**	Implementation of periodic case review meetings (maternal and neonatal deaths and near-miss)
			“*Auditing procedures (following WHO Beyond the Numbers indications) were introduced everywhere and the concept of non-judgmental review of cases understood”*
			*“An audit of maternal and perinatal deaths is carried out every month, with the participation of key staff and hospital managers”*
			*“All partner organizations participating in the assessment contributed to implement Near-Miss Case Review - as a tool to improve the quality of care”*
			*“A blood bank was created as a consequence of maternal death audit”*
Standard 3: Every woman and newborn with condition(s) that cannot be dealt with effectively with the available resources is appropriately referred	Perinatal referral	**+•**	Initial implementation of a referral system for at risk cases and emergency transport made available
			*“Improved and quicker referral of pregnant women in case of complications and emergencies”*
Standard 5: Women and newborns receive care with respect and preservation of their dignity	Mistreatment: detainment, extortion or denial of services.	**++•**	Reduced or cancelled fees for hospital care provision, emergency services and medicines
			Emergency transport made available
			“*The cost exemption policy has significantly reduced barriers to care. Vaginal birth and caesarean section are free of charge. However, women are still expected to bring some materials to the hospital”*

+• − moderate change observed in a minority of countries, +•• − moderate change observed in a majority of countries, ++• − substantial change observed in a minority of countries, ++•• − substantial change observed in a majority of countries

### Factors influencing change, barriers and facilitators

Three studies met the selection criteria to be included in the review of factors influencing change (**Table 6**).

**Table 6:** Key features of studies investigating factors influencing change in quality of MN care

Country (state) and year	Study methods	Sample	Funding agency
Uzbekistan, 2015	Face-to-face interviews	4 hospital directors	UNICEF
Brazil (Pernambuco), 2016	Focus groups plus web-based interviews with key staff	6 hospital directors plus 22 head medical and nursing staff	National Research Council, Brazil
Ethiopia, 2016	Semi-structured face-to-face interviews	1 hospital clinical director, 1 manager and 4 key nursing and medical staff	Doctors with Africa – Cuamm

The first study was carried out in Uzbekistan in 2015 and included 4 regional perinatal centres [14]. The second was carried out in the State of Pernambuco, Brazil, in 2016 and included 6 maternity hospitals [15]. The third was carried out in Ethiopia in 2016 and involved a single district hospital [16]. All three studies were made at the time of reassessment to explore factors influencing change. In the case of Uzbekistan and Brazil, the time elapsed between baseline evaluation and reassessment was one year, whereas in the case of Ethiopia the reassessment occurred after 4 years. All studies were made through interviews with managers and key staff, although with a diversity of methods and sample. **Table 7** and **Table 8** list factors emerging in all three studies or at least in two out of three, as facilitating or hampering improvement of quality. Time constraint was not reported as an obstacle by any of the interviewees. On the contrary, most professionals reported that in order to maintain commitment, the time elapsing between assessment and reassessment should not be too long.

**Table 7:** Internal factors affecting quality improvement

Internal factors that facilitated quality improvement	Internal factors that represented barriers to quality Improvement
• Capacity of the managers and head of units to involve and motivate their staff members (U,E,B)	• High staff turn-over (E,U) and/or fragmentation of staff contracts (B) leading to lack of continuity
• Professional recognition and availability of training and career opportunities as part of the QI action plan (E,B)	• Poor motivation due to lack of professional and monetary incentives (B,E)
• Adequate professional qualification of involved staff and manageable workload (E,B)	• High workload with respect to available human resources (B,E)
• The process of quality assessment itself (U,B)	• Changes in management leading to failure to ensure follow-up to recommendations made in the baseline quality assessment (B,E)

E – Ethiopia, B – Brazil, U – Uzbekistan

**Table 8:** External factors affecting quality improvement

External factors that facilitate change	External factors that represent barriers to change
• Financial and professional incentives provided by partners, donors and government (U,E,B)	• Financial constraints with impact on salaries and equipment (U,E,B)
• Reasonable autonomy at facility level for budget use at facility level (E,B)	• Frequent changes in MoH regulations about human resources and organizational requisites (salaries, working rules) (U,E,B)
• Effective communication with health centres (B,E)	• No result-based professional recognition for staff members involved in QI (U,E,B)

E – Ethiopia, B – Brazil, U – Uzbekistan, QI – quality improvement

## DISCUSSION

To our knowledge this is the first report documenting, in a variety of low and middle income countries and health system settings and by using a homogeneous approach, improvements in the quality of MN hospital care along the continuum of care from admission to discharge. We believe that the review provides several useful indications about the observed improvements and their adherence to WHO standards [11], as well as about key factors influencing change.

Improvements were observed in all areas of care and were particularly important and more frequently observed in the areas corresponding to appropriate case management and respectful care for both mothers and newborns (WHO standards 1 to 6) than in those related to staffing and infrastructure (WHO standards 7 and 8). This is not surprising since changes in the latter areas are more difficult to be achieved by the hospital management and key staff alone, as they usually require action at higher health system levels. Although remarkable and widespread across most facilities and countries, the observed improvements did not cover all the quality gaps that were observed at the baseline assessment nor were they always sufficient to achieve standard care. This could be attributed to the relative shortness of the period elapsing from the baseline assessment to reassessment. However, none of the three studies investigating factors influencing change reported time constraints as a major obstacle, and many assessors involved in reassessment confirmed that change either initiated soon after the assessment or it did not occur. This further underlines the importance of including the development of a draft action plan with timelines and responsibilities to address priority quality gaps as part of the baseline assessment visit, since this allows to use the momentum created by the feedback on quality gaps to create the commitment and identify the individuals who would take on responsibility for it.

While we underline the uniqueness of our findings, in terms of both comprehensiveness of coverage along the continuum of care and variety of settings, we must also recognize their limitations. These derive on one side by the fact that in most countries we were not able to document the details of the process between the two assessments and the extent to which the leading agencies were able to ensure follow-up and support, on the other by the difficulty in disentangling the effects of the inputs provided by the baseline quality assessment from the changes produced by independent factors acting in the same period, such as new laws, regulations and development assistance. However, the very specific features of many of the observed improvements, particularly regarding medical and nursing procedures are hardly explained by inputs other than internal leadership and commitment by involved professionals, and the merit can be attributed with reasonable certainty to the process started through the WHO QA/QI MN tool. In particular, the tool adopts a participatory approach that builds awareness among both managers and staff about the quality gaps and their causes, provides professional support in identifying appropriate practices and providing evidence, and introduces guiding concepts and tools such as the WHO quality of care standards. Indeed, standards cannot be implemented without a learning process that needs professional and contingent inputs along the continuum of care [17], not just bureaucratic recording of what is missing and written protocols of what should be done. The participatory and professional feature of the assessment required by the WHO tool is key to prompt this learning process, as indicated also by the review of the studies on barriers and facilitators. This analysis identified some commonalities across the variety of health system contexts. Among these, the human resource component emerged as crucial, in all its dimensions of manpower availability and deployment, training, continuous professional development and motivation. The capacity of hospital managers and of key professionals, such as unit or department heads, to motivate their staff, and to provide opportunities for professional development emerged as a factor that ultimately made most of the difference at facility level. Although staff numbers, qualification and salaries are obviously important, the issue of human resource management by hospital leading staff is still greatly undervalued, when not neglected. In fact, the differences in the quality of care provided among hospitals belonging to the same system and struggling with the same structural and budgetary difficulties point to the human factor dimension as something which should have a higher place in the health system agendas, and to the need of integrating preservice curricula of all health professionals, from nursing and midwifery staff to managers [18-21]. Based on this evidence, it appears crucial that quality assessments not only provide a snapshot of the existent, but include a dynamic component, namely by initiating the development of a plan of action and defining a time frame for its implementation, as envisaged in the process promoted by the WHO MN tool. Ideally, in addition, prospective identification of barriers and facilitators may help to avoid potential frustration of good will by setting realistic targets for the action plan thus increasing the likelihood of success. A recent review of MN quality assessment tools indeed identified the lack of a clear path leading to action as one of the main pitfalls of the existing tools [9].

In spite of recent progress, the attention paid to quality of care, the capacity to implement quality improvement cycles and take into account patients’ rights and perspectives is still insufficient in all health systems and at all levels [4,9,10]. At facility level, quality assessment tools should promote a constructive dialogue with hospital managers and key staff and build their awareness of quality issues and their capacity of implementing quality cycles also including patients’ views. The review of the experience made with the WHO QA/QI MN tool is encouraging as it shows that this is possible. This refocuses the need of policy changes at national level in support of QI at local level, such as efforts to build capacity for supportive supervision and coaching of auditing processes, including patients’ views: professional staff, managers and patients are all key players of a quality movement [10,22,23].
